# Substrate Versatility of a Recombinant Bifidobacterial Endoglycosidase: *N*-Glycan Release and Profiling from Milk and Plant Glycoproteins

**DOI:** 10.3390/ijms27156633

**Published:** 2026-07-25

**Authors:** Hatice Duman, İzzet Avcı, Bekir Salih, Hacı Mehmet Kayılı, Sercan Karav

**Affiliations:** 1 Department of Molecular Biology and Genetics, Çanakkale Onsekiz Mart University, 17100 Canakkale, Türkiye; hatice.duman@comu.edu.tr; 2 Department of Chemistry, Faculty of Science, Hacettepe University, 06800 Ankara, Türkiye; izzetavci35@gmail.com (İ.A.); bekir@hacettepe.edu.tr (B.S.); 3 Department of Medical Engineering, Faculty of Engineering, Karabük University, 78000 Karabük, Türkiye; h.mehmetkayili@karabuk.edu.tr; 4 Department of Medical Research, China Medical University Hospital, China Medical University, Taichung 406040, Taiwan

**Keywords:** endo*-β-N-*acetylglucosaminidases, deglycosylation, *N*-glycans, lactoferrin, soy protein, *Bifidobacterium infantis*, glycan profiling

## Abstract

*N*-linked glycans (*N*-glycans) are complex carbohydrate structures covalently attached to the asparagine residues of glycoproteins and play a substantial role in protein folding, stability and biological activity. One of the major challenges in glycomics is the enzymatic release of intact *N*-glycans from native glycoproteins, as conventional deglycosylation approaches often require prior protein denaturation and exhibit limited activity toward structurally diverse substrates. In this study, we recombinantly produced EndoBI-1, an endo-*β-N*-acetylglucosaminidase derived from *Bifidobacterium longum subsp. infantis* ATCC 15697, and investigated its *N*-glycan release activity on two structurally distinct glycoprotein substrates: bovine lactoferrin (bLF), an animal-derived glycoprotein, and soy protein, a plant-derived glycoprotein. EndoBI-1 was produced using an *in vivo* cloning approach and purified to homogeneity, as validated by SDS-PAGE analysis. The released *N*-glycan profiles of each substrate were characterized by HILIC-FLD-QTOF-MS/MS analysis, which showed different glycan compositions depending on the origin: complex-type and sialylated *N*-glycans were the major structures in the bLF derived fractions, while the fractions derived from soy protein mainly contained high-mannose-type structures. Together, these results demonstrate the capacity of EndoBI-1 to release structurally intact *N*-glycans from both animal- and plant-derived glycoproteins under native, non-denaturing conditions, establishing this enzyme as a biocatalytic tool for the preparative-scale production of bioactive *N*-glycans from diverse protein sources.

## 1. Introduction

Protein glycosylation is a prevalent and essential post-translational modification that influences multiple facets of proteins. It is essential in determining their structural stability, targeting, folding, and ultimate biological activity [1,2,3]. Glycoproteins are defined by the presence of oligosaccharides (glycans) covalently linked to the polypeptide backbone, which can occur through either *N*-glycosidic or *O*-glycosidic linkages [4]. *O*-linked glycans (*O*-glycans) are conjugated to the hydroxyl groups of serine (Ser) or threonine (Thr) residues through *N*-acetylgalactosamine (GalNAc), resulting in various structurally distinct core classes [5]. *N*-linked glycans (*N*-glycans) are attached to the amide nitrogen of asparagine (Asn) residues within the consensus sequence Asn-X-Ser/Thr (where X ≠ Pro) via *N*-acetylglucosamine (GlcNAc) [6]. All *N*-glycans possess a conserved core structure consisting of two GlcNAc residues followed by three mannose (Man) residues, which can be further modified to produce high-mannose, hybrid, or complex-type glycans [7]. High mannose (HM) glycans typically have five to nine Man residues branching from the chitobiose core, while hybrid and complex glycans integrate additional GlcNAc, galactose (Gal), fucose (Fuc), and sialic acid (NeuAc) residues in diverse branching configurations [8]. On the other hand, animal-derived glycoproteins predominantly carry complex-type *N*-glycans decorated with sialic acid, galactose, and core fucose residues [4,7]. In contrast, plant-derived glycoproteins are characteristically modified with β(1,2)-xylose and α(1,3)-fucose residues, structural features absent in mammalian *N*-glycans. These fundamental differences in *N*-glycan architecture between animal and plant sources have important implications for enzyme-substrate interactions, glycan bioavailability, and downstream biological activity [9,10].

The structural diversity of *N*-glycans confirms their extensive biological roles and underscores the necessity for analytical and enzymatic methods that can selectively release and characterize these structures [4,11,12]. Commonly used deglycosylation methods are mainly based on peptide-*N*-glycosidase F (PNGase F), an amidase that cleaves the amide link between the innermost GlcNAc and asparagine residues [13]. However, PNGase F has some well-established limitations. It requires prior protein denaturation to work efficiently on folded glycoproteins, does not work on core a(1,3)-fucosylated *N*-glycans frequently recognized in plant and invertebrate glycoproteins, and yields deaminated asparagine residues rather than intact glycopeptides [14]. Other commercial endoglycosidases such as Endo H, Endo F1, F2, and F3 show better action on native glycoproteins than PNGases, but limited activity on multi-antennary complex-type *N*-glycans [15,16]. Endoglycosidases cleave within the chitobiose core of *N*-glycans, specifically the β(1,4)-glycosidic bond between the two innermost GlcNAc residues, releasing intact *N*-glycans with a single GlcNAc moiety connected to the protein, and are able to function on native, non-denatured glycoproteins [14].

Among the characterized endoglycosidases, EndoBI-1, an endo-*β-N*-acetylglucosaminidase originally identified in *Bifidobacterium longum subsp. infantis* (*B. infantis*) ATCC 15697, has emerged as a biocatalytic tool for *N*-glycan release. EndoBI-1 is a member of the glycoside hydrolase family 18 (GH18) and cleaves the β(1,4)-glycosidic bond within the *N-N′-*diacetylchitobiose core of *N*-glycans, releasing intact oligosaccharide structures with a single GlcNAc residue remaining on the protein backbone [17,18]. In contrast to the structurally related EndoBB from *B. longum* DJO10A, which is restricted to high-mannose-type substrates, EndoBI-1 shows broad substrate specificity, cleaving high-mannose, hybrid and complex *N*-glycans, including core-fucosylated structures resistant to deglycosylation by PNGase F and other commercial endoglycosidases [17,19]. The extensive substrate specificity, together with the demonstrated thermostability at 95 °C and the ability to act on native, non-denatured glycoproteins under mild reaction conditions, renders EndoBI-1 a promising candidate for both analytical and industrial applications [20].

*In silico* approaches have been used to do structural investigations of EndoBI-1 to provide vital insights into the molecular basis of its activity and substrate selectivity. Comparative homology modeling and molecular docking studies of the +1 and +1′ subsites indicated that EndoBI-1 contains a transmembrane helix domain anchoring it to the bacterial cell membrane and that the substrate-binding architecture is structurally different from that of EndoBI-2, an isoenzyme also encoded in the *B. infantis* genome. These structural properties are consistent with the preference of EndoBI-1 for milk-derived glycoproteins and its improved identification of complex-type *N*-glycan structures in animal-derived substrates [21].

Recombinant production of EndoBI-1 in *Escherichia coli* (*E. coli*) has been established and validated, providing a scalable and reproducible enzyme preparation for downstream applications [17]. The enzyme’s activity on milk-derived glycoprotein substrates has been previously reported, including model glycoproteins RNaseB and bovine lactoferrin (bLF) and concentrated bovine colostrum whey, indicating its broad action on animal-derived glycoproteins [17,22]. EndoBI-1 immobilization in several formats kept full *N*-glycan release specificity, further demonstrating its potential for scalable industrial applications [23]. The *N*-glycans released from milk glycoproteins by EndoBI-1 have been demonstrated to serve as selective growth substrates for *B. infantis*, supporting their potential as functional prebiotic ingredients [24].

One of the most studied substrates for EndoBI-1 is bLF. Lactoferrin is a multifunctional iron-binding glycoprotein (~80 kDa) which is abundant in bovine colostrum and milk and has antibacterial, antiviral, anti-inflammatory and immunomodulatory effects [25]. bLF may have up to five *N*-glycosylation sites, most of which are filled with complex-type, sialylated *N*-glycans, with a small amount of high-mannose structures [26]. The glycan moieties of lactoferrin are not structural decorations but have been proven to directly contribute to its antibacterial effectiveness, proteolytic resistance and receptor binding ability [25]. Selective enzymatic deglycosylation of lactoferrin using EndoBI-1 has been previously applied to study the role of certain classes of glycans in the biological activities of lactoferrin, highlighting the importance of *N*-glycan composition in lactoferrin function [20].

Soybean protein is an important plant protein around the world. The increasing need for sustainable protein sources has highlighted soy protein as a functional food component [27]. The plant-based protein market is expected to develop significantly over the next decade [28,29]. Glycinin (11S) and β-conglycinin (7S) are the main storage proteins of soybean. These two proteins are glycoproteins with a glycosylation profile mostly consisting of high-mannose type *N-*glycans, which is typical for plant-derived proteins [30,31]. While the activity of EndoBI-1 on animal-derived substrates has been extensively described, its capacity to release *N*-glycans from plant-derived glycoproteins remains largely unexplored. The present work addresses this gap for a plant-derived substrate, soy protein.

In the present research, we recombinantly produced EndoBI-1 and examined its *N*-glycan release activity on two structurally different substrates, bLF as a model animal-derived glycoprotein and soy protein as a model plant-derived glycoprotein. Enzymatic activity was assessed on both substrates, and the released *N*-glycans were subsequently profiled using HILIC-FLD-QTOF-MS/MS. The comparison of the glycan compositions released from these two structurally distinct substrates contributes to the growing understanding of endoglycosidase-mediated *N*-glycan release from both animal and plant sources.

## 2. Results

### 2.1. Recombinant Production and Activity Assessment of EndoBI-1

**Figure 1:**
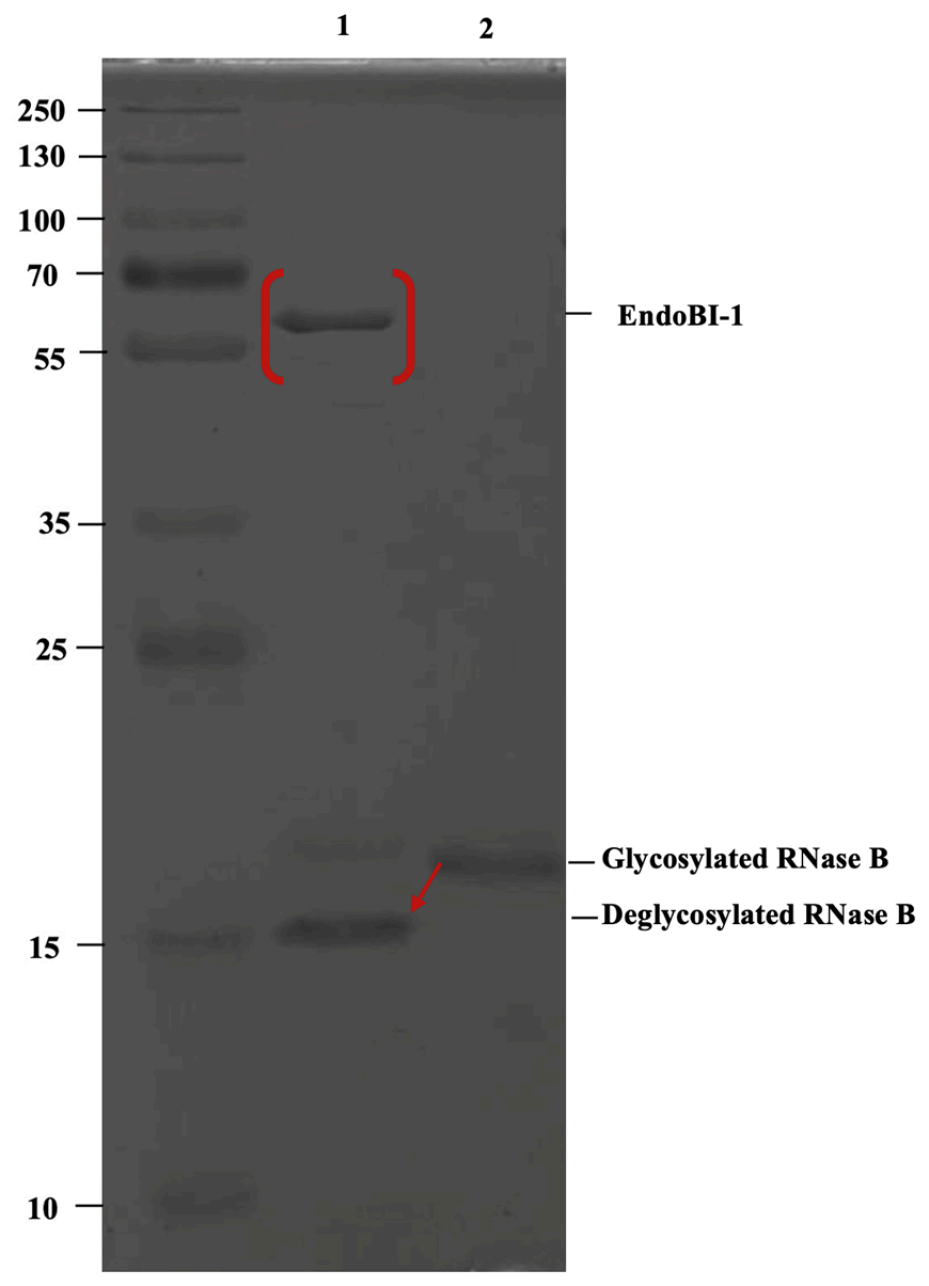
SDS-PAGE (4-12%) analysis of recombinant EndoBI-1 production and verification of enzymatic activity on model glycoprotein RNase B. Lane 1: Purified recombinant EndoBI-1 (~55.94 kDa) and deglycosylated RNase B (~14 kDa). Lane 2: Glycosylated RNase B (17 kDa). Samples were denatured in Laemmli sample buffer at 95 °C for 5 min prior to loading. Gels were stained with Coomassie Brilliant Blue.

Recombinant EndoBI-1 was successfully expressed in *E. cloni* 10G cells under L-rhamnose induction using the pRham N-His Kan vector and purified by nickel-affinity chromatography. SDS-PAGE analysis confirmed the presence of a prominent band at approximately 56 kDa, consistent with the theoretical molecular weight of EndoBI-1 including the N-terminal 6xHis affinity tag, indicating successful expression and purification (Figure 1, Lane 1). A total of 7 mg of purified EndoBI-1 was obtained from 50 mL of culture media and stored at −80 °C until further use.

To evaluate the enzyme activity, recombinant EndoBI-1 was incubated with ribonuclease B (RNase B), a model glycoprotein with only high-mannose type *N*-glycans at a final enzyme concentration of 0.025 mg/mL. Following incubation at 37 °C in 0.02 M Na_2_HPO_4_ buffer at pH 5, SDS-PAGE analysis showed a downward band shift in RNase B from 17 kDa to ~14 kDa, confirming successful cleavage of the chitobiose core and release of *N*-glycan (Figure 1).

### 2.2. Deglycosylation of bLF by EndoBI-1 and N-Glycan Profiling

**Figure 2:**
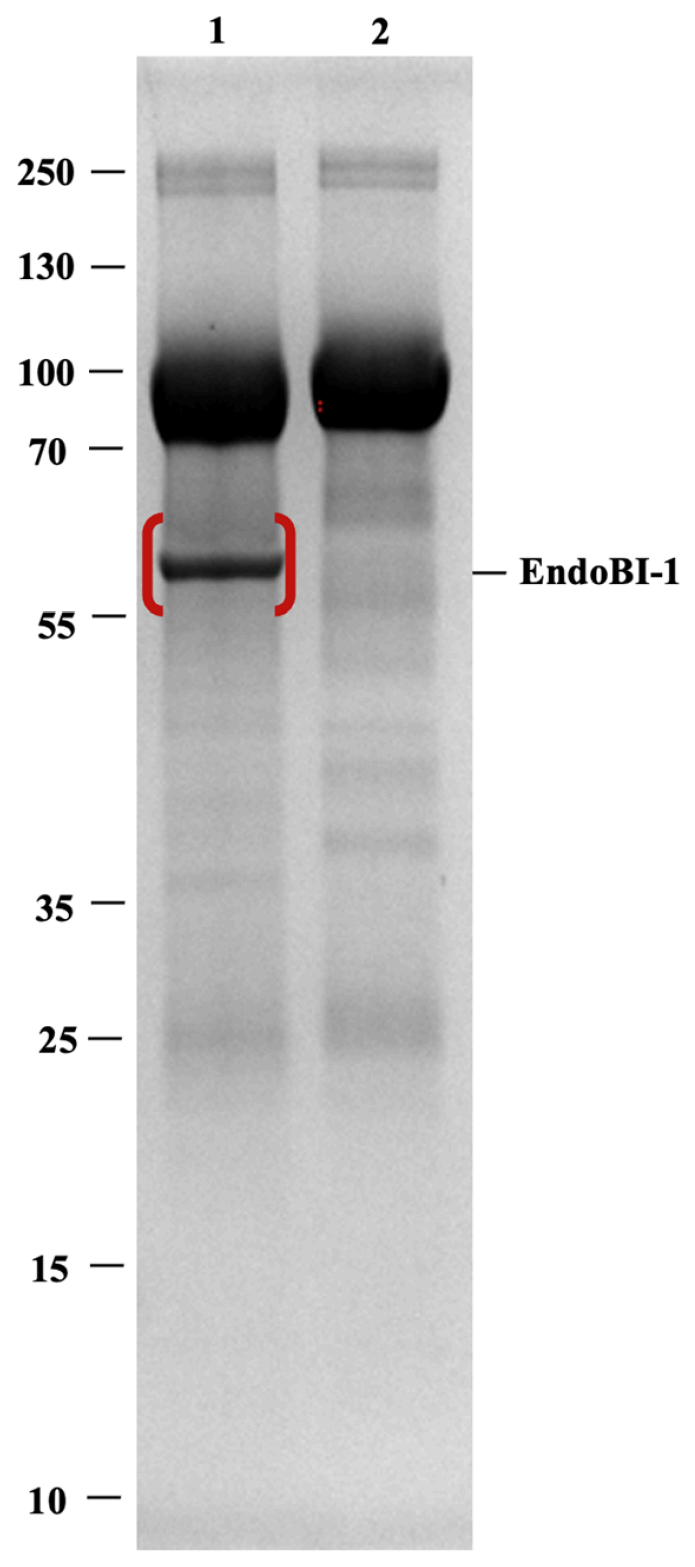
SDS-PAGE (4–12%) analysis of enzymatic deglycosylation of bLF by recombinant EndoBI-1. Lane 1: Deglycosylated bLF treated with EndoBI-1 (~55.94 kDa). Lane 2: Glycosylated bLF control (~80 kDa).

SDS-PAGE analysis of the EndoBI-1-treated bLF confirmed successful enzymatic deglycosylation under native, non-denaturing reaction conditions (Figure 2). EndoBI-1 (~55.94 kDa) was observed in Lane 1 alongside the bLF band, while Lane 2 showed the intact glycosylated bLF control at ~80 kDa. The gel is presented as qualitative confirmation of enzyme presence and substrate integrity. Definitive evidence of *N*-glycan release was obtained by HILIC-FLD-QTOF-MS/MS analysis.

**Figure 3:**
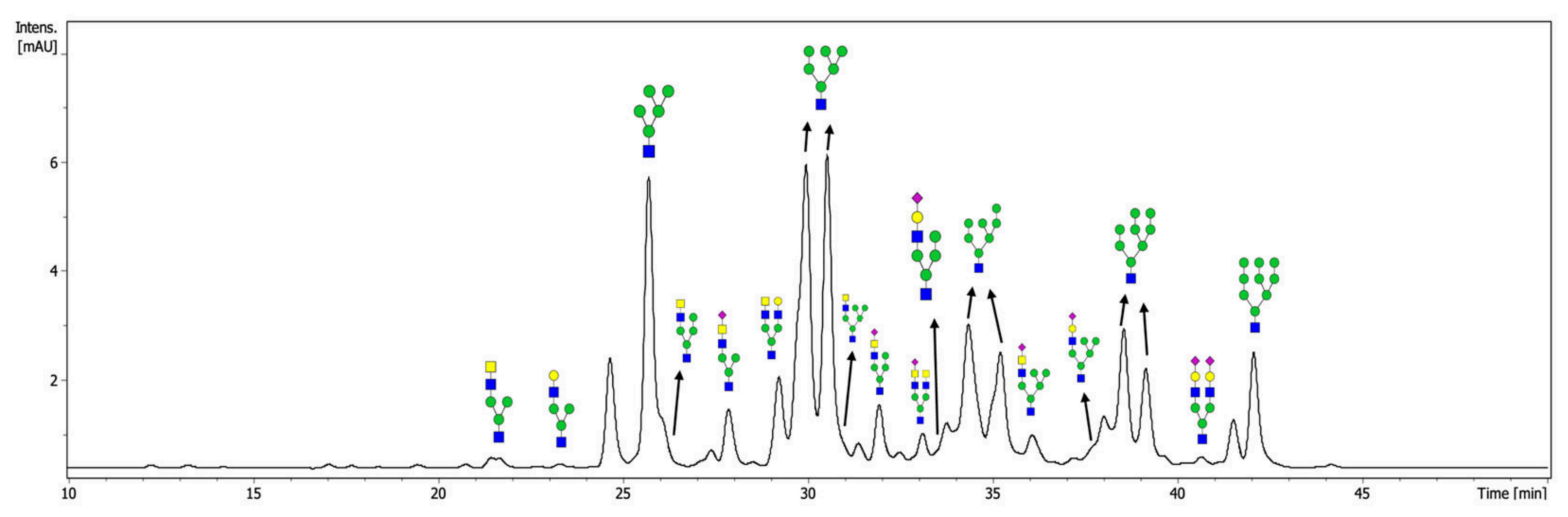
HILIC-FLD chromatogram of procainamide-labeled *N*-glycans released from bLF by recombinant EndoBI-1. Identified *N*-glycan structures are indicated by arrows and annotated above the corresponding chromatographic peaks. *N*-glycan structures were determined based on their MS/MS fragmentation patterns and annotated using GlycoWorkBench software 2.0 with reference to a glycan library containing common *N*-glycan monosaccharides. Monosaccharide residues are depicted according to the Symbol Nomenclature for Glycans (SNFG) system: green circle, mannose (Man); blue square, *N*-acetylglucosamine (GlcNAc); yellow circle, galactose (Gal); yellow square, *N*-acetylgalactosamine (GalNAc); pink diamond, *N*-acetylneuraminic acid (Neu5Ac); proc, procainamide label attached to the reducing end of each glycan structure. The chromatogram corresponds to a pooled sample derived from three independent deglycosylation replicates.

Following deglycosylation, released *N*-glycans were labeled with procainamide and analyzed by HILIC-FLD-QTOF-MS/MS. To process raw LC-QTOF-MS/MS data, the GlycoQuest algorithm was first applied, using the ProteinScape software V4 (Bruker Daltonics). A search of the CarbBank database was carried out using procainamide as the reducing end modification (Figure 3) [32].

**Figure 4:**
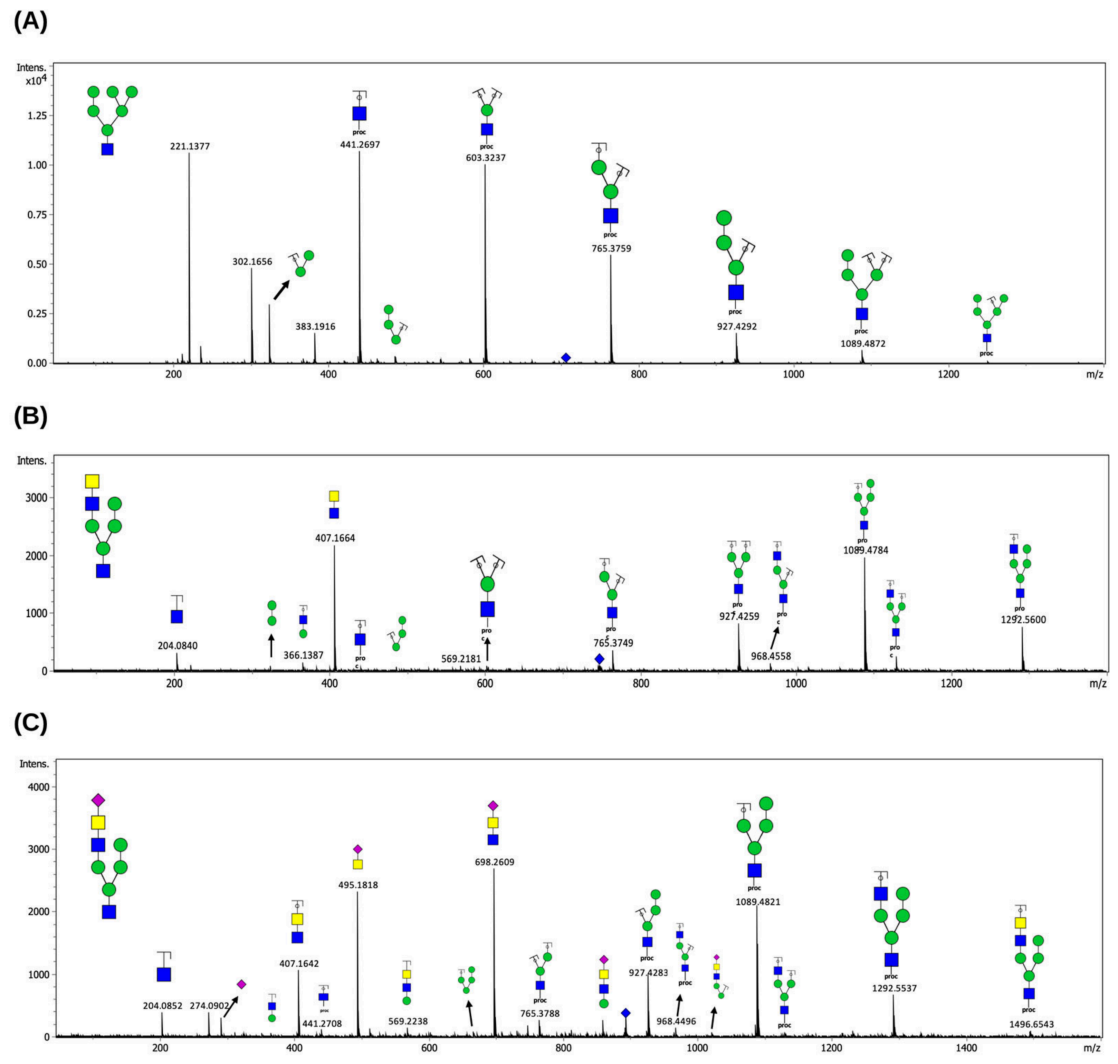
MS/MS spectra of *N*-glycans released from bLF by recombinant EndoBI-1. Panels (**A**–**C**) present representative MS/MS spectra of major *N*-glycan types identified in bLF. The detected structures correspond to high-mannose-type *N*-glycan H6N1 (Man6GlcNAc) (**A**); complex-type *N*-glycan H4N3 (Man4GlcNAc3) (**B**); and sialylated complex-type *N*-glycan H4N3S1 (Man4GlcNAc3NeuAc) (**C**). Peak annotation and structural assignment were performed using GlycoWorkBench software. Green circles, yellow circles, blue squares, yellow squares, and pink diamonds represent Man, Gal, GlcNAc, GalNAc, and NeuAc residues, respectively. MS/MS spectra correspond to a pooled sample derived from three independent deglycosylation replicates.

A total of 17 distinct *N*-glycan compositions were identified from bLF by MS/MS fragmentation and annotated using GlycoWorkBench software (Table 1, Figure 4). These compositions encompassed three structural classes: five high-mannose-type *N*-glycans (H5N1, H6N1, H7N1, H8N1, H9N1), five complex-type *N*-glycans (H3N3, H4N2, H4N3, H4N4, H5N3), and seven sialylated *N*-glycans (H3N3S1, H4N3S1, H3N5S1, H5N2S1, H5N3S1, H6N2S1, H5N3S2).

### 2.3. Deglycosylation of Soy Protein by EndoBI-1 and N-Glycan Profiling

**Figure 5:**
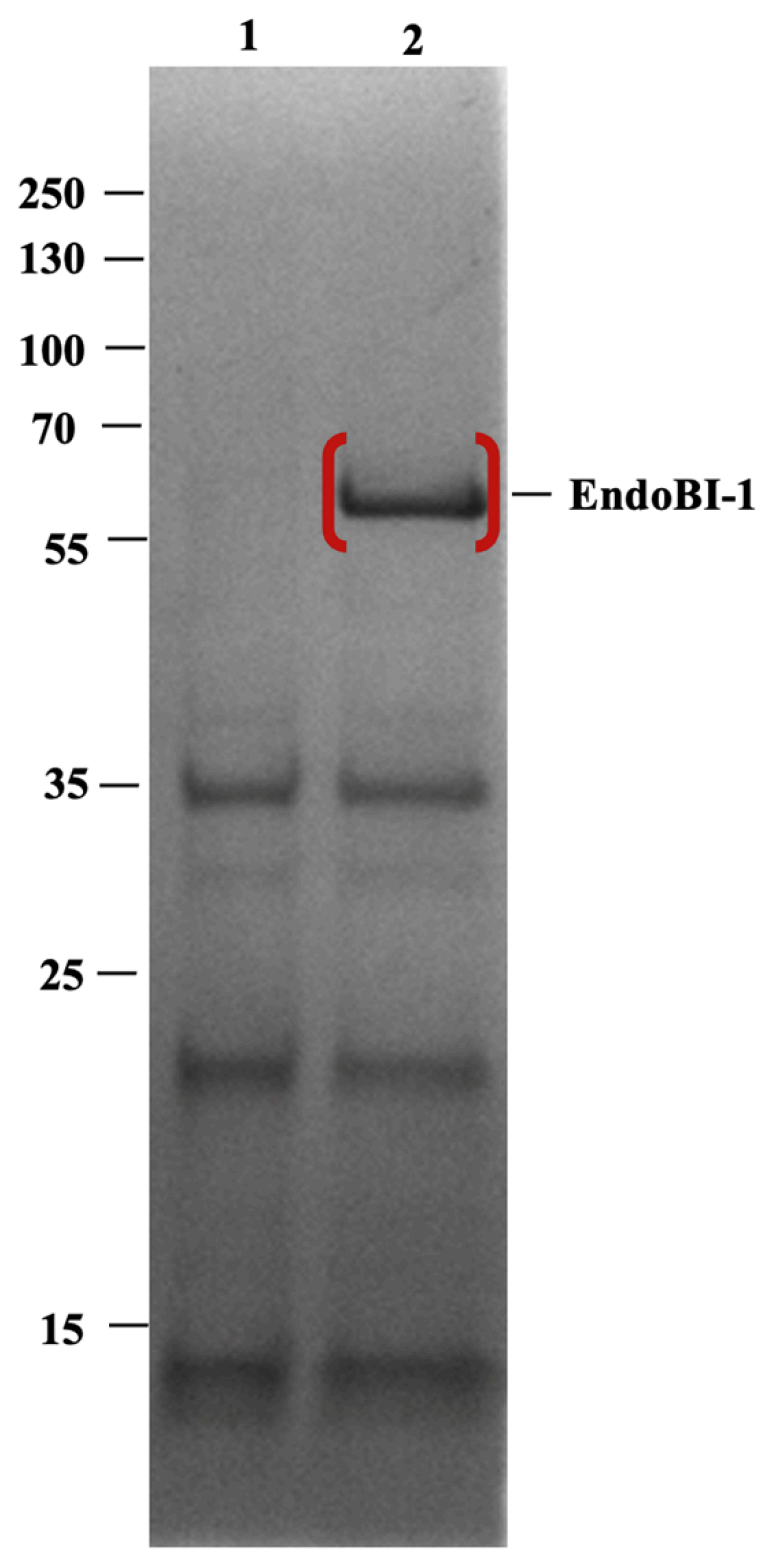
SDS-PAGE (4–12%) analysis of enzymatic deglycosylation of soy protein by recombinant EndoBI-1. Lane 1: Glycosylated soy protein control. Lane 2: Deglycosylated soy protein treated with EndoBI-1 (~55.94 kDa). The gel is presented as qualitative confirmation of enzyme presence and substrate integrity. Definitive evidence of *N*-glycan release was obtained by HILIC-FLD-QTOF-MS/MS analysis.

SDS-PAGE analysis confirmed enzyme presence and substrate integrity following EndoBI-1 treatment of soy protein under native, non-denaturing reaction conditions (Figure 5). Lane 1, intact glycosylated soy protein control. Lane 2, EndoBI-1 (~56 kDa) and deglycosylated soy protein bands appeared demonstrating that enzymatic cleavage of *N*-glycans from the soy protein substrate was active. The gel is presented as qualitative confirmation of enzyme presence and substrate integrity. Definitive evidence of *N*-glycan release was obtained by HILIC-FLD-QTOF-MS/MS analysis.

**Figure 6:**
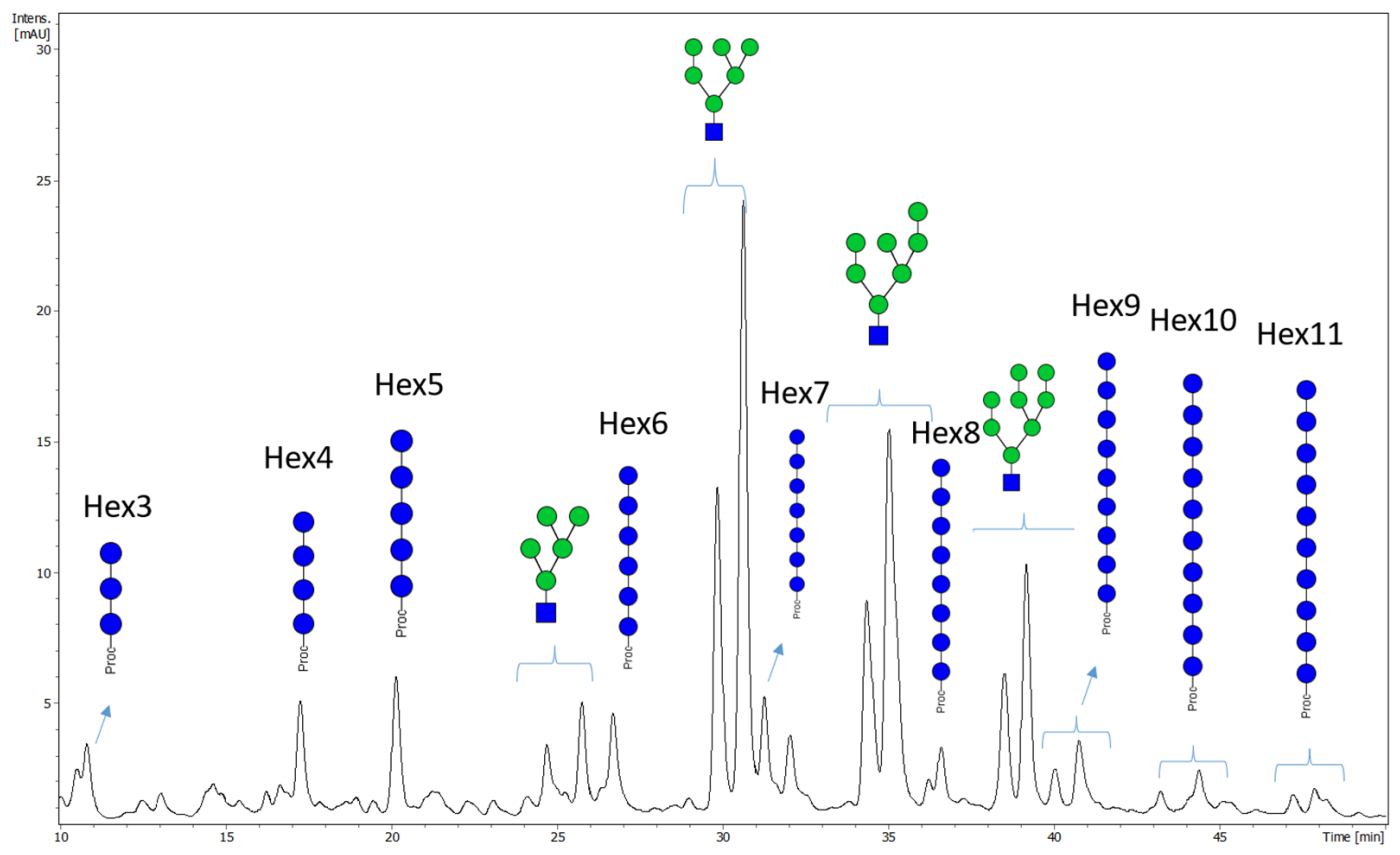
HILIC-FLD chromatogram of procainamide-labeled *N*-glycans released from soy protein by recombinant EndoBI-1. Peak annotation and structural assignment were performed using GlycoWorkBench software. Green circles, blue squares, and blue circles represent mannose (Man), *N*-acetylglucosamine (GlcNAc), and glucose (Glc) residues, respectively. The chromatogram corresponds to a pooled sample derived from three independent deglycosylation replicates.

The HILIC-FLD chromatogram of soy protein-derived *N-*glycans revealed a markedly simpler elution profile compared to that of bLF, with a limited number of well-resolved peaks eluting within a narrow retention time window (Figure 6).

**Figure 7:**
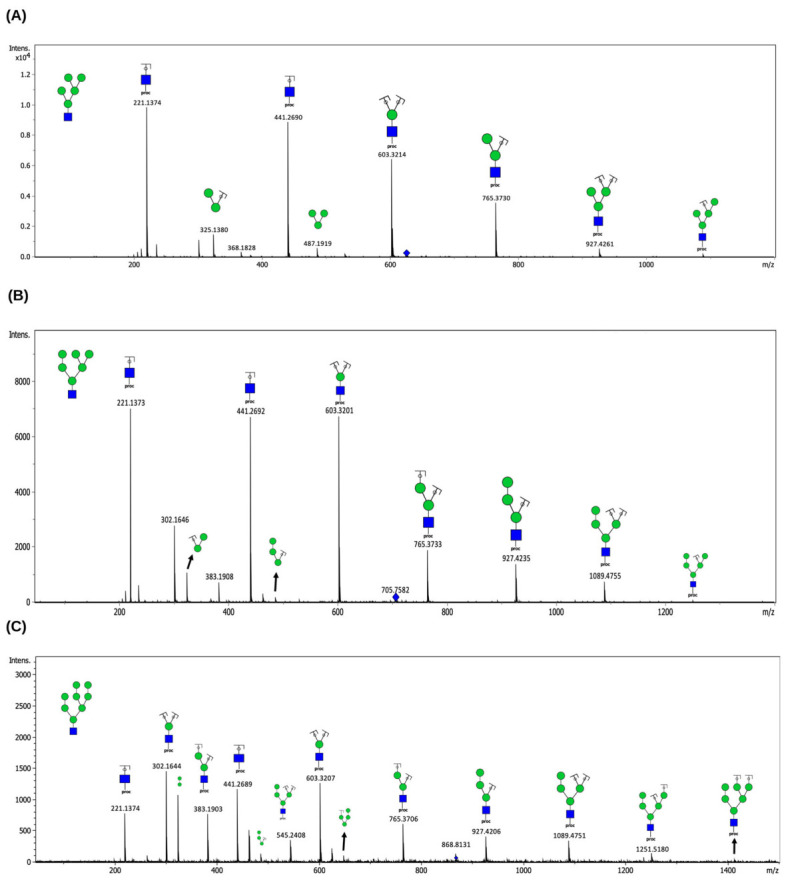
MS/MS spectra of *N*-glycans released from soy protein by recombinant EndoBI-1. Panels (**A**–**C**) present representative MS/MS spectra of high-mannose-type *N*-glycans identified in soy protein. The detected structures correspond to H5N1 (Man5GlcNAc) in panel (**A**); H6N1 (Man6GlcNAc) in panel (**B**); and H8N1 (Man8GlcNAc) in panel (**C**). Peak annotation and structural assignment were performed using GlycoWorkBench software. Green circles and blue squares represent Man and GlcNAc residues, respectively. MS/MS spectra correspond to a pooled sample derived from three independent deglycosylation replicates.

MS/MS fragmentation analysis identified four distinct *N*-glycan compositions from soy protein: H5N1 (Man5GlcNAc), H6N1 (Man6GlcNAc), H7N1 (Man7GlcNAc), and H8N1 (Man8GlcNAc) (Table 1, Figure 7). All identified structures were exclusively of the high-mannose type, with no complex-type, hybrid, sialylated, or fucosylated *N*-glycans detected.

The *N*-glycan compositions identified from both substrates are summarized in Table 2.

## 3. Discussion

### 3.1. Recombinant Production and Activity of EndoBI-1

The recombinant production of EndoBI-1 in the present study yielded 7 mg of purified protein from 50 mL of culture media, representing a notable improvement over previously reported yields for this enzyme. This yield compares favorably with previously reported recombinant EndoBI-1 productions, where yields of approximately 1.7 mg/L and 1.34 mg/100 mL were obtained using IPTG-inducible expression systems in *E. coli* BL21 [17,22]. The improved yield observed in the present study may be attributed to the use of the rhaPBAD promoter-based Expresso Rhamnose expression system, which enables tunable induction levels and favors the accumulation of soluble, active protein compared to conventional IPTG-based systems [33]. The transmembrane and signal peptide regions were excluded from the construct to facilitate soluble cytoplasmic expression, in accordance with previously reported structural insights for this enzyme [21]. The observed band shift in RNase B from 17 kDa to ~14 kDa confirms the catalytic integrity of the recombinant enzyme preparation, consistent with the established quality-control protocol for this assay [17,22].

### 3.2. N-Glycan Profile of bLF Released by EndoBI-1

The ability of EndoBI-1 to act on native, non-denatured bLF is consistent with its previously reported activity on native glycoproteins under mild reaction conditions [17,22]. This is particularly relevant for applications where preservation of the native protein structure is essential, such as investigations addressing the individual biological contributions of glycans and the protein backbone [22]. It is noted that EndoBI-1 hydrolyzes the β(1,4) bond within the chitobiose core, leaving the innermost GlcNAc attached to the polypeptide. For bLF, which carries five *N*-glycosylation sites, complete release corresponds to a theoretical mass loss of approximately 6 to 9 kDa from an 80 kDa protein; shifts of this magnitude are not reliably resolved on a 4-12% gel. The definitive evidence of *N*-glycan release is therefore provided by the HILIC-FLD-QTOF-MS/MS data.

The structural diversity of the released *N*-glycan pool is consistent with the well-established glycosylation heterogeneity of bLF, which carries up to five *N*-glycosylation sites with a mixture of high-mannose, hybrid, and complex-type structures [25].

The identification of high-mannose-type *N-*glycans ranging from H5N1 to H9N1 is in agreement with published bLF glycoprofiles, in which oligomannose-type structures constitute the predominant fraction, accounting for approximately 65% of total *N*-glycans in bLF [22]. Previous site-specific glycoproteomic studies have demonstrated that high-mannose structures are particularly enriched at specific glycosylation sites of bLF, including Asn252 and Asn564, with Man9GlcNAc2 reported as one of the most abundant high-mannose forms in bLF [26]. The detection of Man5 through Man9 series in our analysis is fully consistent with these reports, confirming that EndoBI-1 accesses and cleaves the chitobiose core of high-mannose *N*-glycans under native conditions.

The absence of fucosylated *N*-glycan structures in the released pool is comparable with the previously described glycosylation profile of commercial bLF, which is dominated by high-mannose and sialylated complex-type structures with low core fucosylation. It is important to note that the ability of EndoBI-1 to cleave core-fucosylated *N*-glycans, structures resistant to PNGases, has been well established [14]. This indicates that the lack of fucosylated structures in the present dataset reflects the glycosylation profile of the substrate rather than an enzymatic limitation of the enzyme.

Comparison with previous reports demonstrates that the *N*-glycan diversity released by EndoBI-1 from native bLF in the present study is consistent with, albeit more limited in number than those reported by Bunyatratchata et al. [22] who released 32 distinct *N*-glycan compositions from native bovine colostrum whey glycoproteins, a complex mixture containing lactoferrin, immunoglobulins and other glycoproteins. The smaller number of recognized structures observed in the present study could be due to the fact that a single pure glycoprotein substrate was used rather than a heterogeneous mixture of whey protein, which would intrinsically have limited glycan structural diversity. Moreover, the analytical platform variations, HILIC-FLD-QTOF-MS/MS with procainamide labeling in the present investigation compared to nano-LC-Chip-Q-TOF in prior findings, may contribute to the disparities in glycan coverage and detection sensitivity [32].

### 3.3. N-Glycan Profile of Soy Protein Released by EndoBI-1

The soy protein used in this study mainly consisted of two major soybean storage proteins: β-conglycinin (7S globulin), a trimeric glycoprotein with a molecular weight of 150–200 kDa, having α’ (~72 kDa), α (~68 kDa) and β (~52 kDa) subunits, and glycinin (11S globulin), a hexameric protein with a molecular weight of 300-380 kDa. Of these, β-conglycinin is the predominant glycoprotein portion, with *N*-glycans at five asparagine residues on its three subunits, Asn199 and Asn455 on the α subunit, Asn215 and Asn471 on the α’ subunit and Asn328 on the β subunit [30,34]. The ability of EndoBI-1 to act on native soy protein without prior denaturation is consistent with its previously reported activity under mild, non-denaturing reaction conditions. Conventional enzymatic approaches have been reported to exhibit limited activity on plant-derived glycoproteins due to the presence of core α(1,3)-fucose residues in many plant glycoproteins [14]. For the β-conglycinin subunits (72, 68 and 52 kDa, each carrying one to two glycans), complete *N*-glycan release corresponds to a theoretical mass loss of approximately 1 to 3 kDa, which is below the reliable resolution limit of a 4-12% SDS-PAGE gel. The definitive evidence of *N*-glycan release is therefore provided by the HILIC-FLD-QTOF-MS/MS data.

Deglycosylation was followed by labeling of the liberated *N*-glycans with procainamide and analysis by HILIC-FLD-QTOF-MS/MS. The HILIC-FLD chromatogram of *N*-glycans produced from soy protein was significantly simpler than that of bLF with a few well-resolved peaks eluting in a short retention time range (Figure 6). Interestingly, apart from enzyme-released *N*-glycans, the chromatogram obtained by HILIC-FLD also showed a series of low-molecular weight hexose oligomers (Hex3 to Hex11), presumably originating from the endogenous soy oligosaccharides that were co-extracted during the protein extraction process. This *N*-glycan analytical workflow enabled the simultaneous detection of both enzymatically released *N*-glycans and naturally occurring soluble oligosaccharides within the same sample. The simplicity of the chromatogram is compatible with a structurally homogeneous high-mannose type glycosylation profile, which is typical for plant storage glycoproteins and lacks the diversity of complex-type and sialylated *N*-glycans found in animal glycoproteins [9,30,35]. Importantly, the selectivity of the chromatographic separation was excellent on the HILIC platform; branched high-mannose N-glycans were completely resolved from the free linear glucose oligomers (Hex3-Hex11) identified in the same sample.

The high-mannose-type *N*-glycan prevalence in soy protein is extensively documented in the literature. High-mannose-type *N*-glycans with glycoforms from Man5 to Man9 structures were exclusively found on the soybean allergen β-conglycinin as demonstrated by Li *et al.* [30]. Liew et al. [36] characterized high-mannose *N*-glycans from 8 different bean species including soybean in detail using state-of-the-art tandem mass spectrometry and identified Man5 through Man9 isomers as the dominant species across all species studied and proposed soybean as a feasible natural source for large-scale high-mannose *N*-glycan production. Furthermore, Bolino et al. profiled the *N*-glycomes of common dietary protein sources, such as soybean, egg white, pea and bovine whey and showed that oligomannose-type *N*-glycans are exclusively enriched in plant-derived proteins, in contrast to the complex and sialylated *N*-glycan profiles that are typical of animal-derived proteins [35]. It should be noted that the soy protein used in this study was not subjected to subunit-specific characterization; the glycosylation status of individual proteins such as glycinin and β-conglycinin was therefore not separately assessed. However, the exclusive detection of high-mannose *N*-glycans (Man5-Man8) across the entire isolate, with no complex, hybrid, or sialylated structures, indicates that the global *N*-glycome of this matrix is overwhelmingly restricted to high-mannose structures, regardless of the precise subunit composition. Future targeted glycoproteomic studies would be required to assign these structures to individual protein subunits.

From a structural point of view, it is interesting to note that only high-mannose *N*-glycans were detected in the soy protein, with no β(1,2)-xylose or α(1,3)-fucose-containing structures. These are plant-specific changes, generally seen in plant-derived glycoproteins, which are cross-reactive carbohydrate determinants (CCDs) involved in food allergenicity and IgE-mediated immune recognition [30]. The absence of these residues in the liberated *N*-glycan pool most probably reflects the high-mannose type glycosylation of β-conglycinin, where these plant epitopes are not found on the core high-mannose structures. Furthermore, the lack of core α(1,3)-fucose on the released high-mannose *N*-glycans is in agreement with their susceptibility to cleavage by EndoBI-1, which cleaves the β(1,4) bond between the two core GlcNAc residues, a site accessible irrespective of antennary fucosylation patterns [17].

To our knowledge, this is the first systematic characterization of *N*-glycans released from soy protein by EndoBI-1 using HILIC-FLD-QTOF-MS/MS under native reaction conditions. The high-mannose *N*-glycans identified from soy protein represent candidate structures for future investigation of their prebiotic potential and immunomodulatory properties, as previously reported for structurally related glycans from other sources [30].

### 3.4. Comparative Analysis of N-Glycan Compositions Released from Animal- and Plant- Derived Glycoproteins

EndoBI-1 released a structurally diverse pool from bLF, consisting of 17 different *N*-glycan compositions encompassing high-mannose (n = 5), complex (n = 5), and sialylated (n = 7) structures. In contrast, the soy protein substrate yielded four compositions, all of the high-mannose type (Man5 to Man8), with no complex, hybrid, sialylated, or fucosylated species detected (Table 1 and Table 2). This difference in compositional diversity reflects the fundamentally distinct *N*-glycosylation machinery of animal and plant systems rather than differential enzymatic accessibility [37].

These different profiles result from the differences between animals and plants in *N*-glycan biosynthesis and processing pathways [38]. In mammalian systems, local Golgi enzymes extensively modify the *N*-glycans to form antennary GlcNAc branching, galactosylation, core fucosylation, and terminal sialylation, resulting in the complex- and sialylated-type structures that dominate the bLF pool [4,39]. In contrast, plant storage glycoproteins such as β-conglycinin are mainly decorated with oligomannosidic *N*-glycans, and where complex-type plant *N*-glycans are formed, they are typically modified with β(1,2)-xylose and core α(1,3)-fucose, plant-specific cross-reactive carbohydrate determinants that were not found in the structures released here [40]. The exclusive detection of high-mannose forms in the soy fraction is therefore consistent with the known oligomannosidic nature of soybean storage-protein glycosylation and does not indicate any limitation of EndoBI-1 activity.

This pattern closely resembles that reported by Bolino et al. [35], who demonstrated that animal sources carried diverse complex, hybrid, and sialylated structures, while plant sources were exclusively oligomannosidic. The present results replicate this kingdom-dependent pattern using EndoBI-1 on native, non-denatured substrates, suggesting that, for the two substrates tested, EndoBI-1 faithfully reflects the intrinsic *N*-glycome of a given substrate, consistent with the previously documented substrate tolerance of this enzyme across several glycoprotein substrates [14]. Notably, the bLF and soy *N*-glycan profiles reported here are in agreement with those independently obtained using mechanistically distinct amidase-based release chemistries, as reported by Bolino et al. [35], Li et al. [30], and Liew et al. [36], further supporting that the released glycan repertoires reflect the intrinsic substrate glycomes.

Furthermore, within the present dataset, the bLF reaction serves as an internal control: using the same enzyme preparation, enzyme-to-substrate ratio, buffer and incubation conditions, EndoBI-1 released complex-type and sialylated glycans from bLF in the same experimental series in which only high-mannose structures were obtained from soy protein. The absence of complex-type structures in the soy pool therefore cannot be attributed to a general inability of the enzyme or the analytical workflow to release or detect such structures. The two released glycan pools differ in their expected biological relevance, as reported in the literature. The sialylated and complex-type structures from bLF have been associated with antimicrobial and prebiotic properties in previous studies [24]. The high-mannose structures from soy protein are recognized ligands for mannose-binding lectins and C-type lectin receptors and represent an underexplored, plant-derived candidate pool for future functional investigation, as suggested by previous reports [36]. These findings highlight that a single biocatalyst can provide structurally defined *N*-glycan pools from both animal and plant glycoproteins under mild native conditions.

While the present study provides the first systematic characterization of EndoBI-1 activity on a plant-derived glycoprotein substrate, certain aspects warrant further investigation. Quantitative deglycosylation efficiency and site occupancy could not be determined from released-glycan profiling alone and would require site-specific glycopeptide analysis. As deglycosylation replicates were pooled prior to MS analysis to ensure sufficient material for reliable structural confirmation of lower-abundance compositions, the present dataset provides a qualitative compositional survey rather than a quantitative glycomic profile. Quantitative glycomic profiling with replicate-level MS analysis and internal standards represents a valuable extension of the present work. A direct experimental comparison of EndoBI-1 with commercial deglycosylation enzymes under identical conditions represents a natural extension of the present work and is identified as the subject of dedicated follow-up studies.

## 4. Materials and Methods

### 4.1. Materials and Reagents

bLF from bovine colostrum was purchased from Sigma-Aldrich (St. Louis, MO, USA). Fresh soybeans (Glycine max) were obtained from a local supplier and used for protein isolation as described in Section 4.2. The pRham N-His Kan vector and the Expresso Rhamnose Cloning and Expression System, including *E. cloni* 10G chemically competent cells, were obtained from Lucigen Corporation (Middleton, WI, USA). HisPur Ni-NTA Resin for affinity chromatography was purchased from Thermo Scientific (Waltham, MA, USA). Protein concentrations were determined using the Qubit Protein Assay Kit (Thermo Fisher Scientific, Waltham, MA, USA). For *N*-glycan labeling, procainamide hydrochloride and picoline borane were obtained from Sigma-Aldrich (St. Louis, MO, USA). Acetonitrile (ACN; HPLC grade), ammonium formate, dimethyl sulfoxide (DMSO), glacial acetic acid, imidazole, sodium phosphate dibasic (Na_2_HPO_4_), and all other analytical-grade reagents were purchased from Sigma-Aldrich unless otherwise stated.

### 4.2. Glycoprotein Substrate Preparation

bLF was dissolved in distilled water at the desired working concentration prior to deglycosylation experiments. Soy protein was isolated from fresh Glycine max beans by cryogenic grinding in liquid nitrogen, followed by aqueous extraction in distilled water. Insoluble debris was removed by centrifugation (4000 rpm, 4 °C, 15 min). Protein was then precipitated by two rounds of ice-cold ethanol addition (4:1, *v*/*v*), each followed by centrifugation (4000 rpm, 4 °C, 15 min), with modifications to a previously described protocol [35]. The recovered protein pellet was resuspended in distilled water and further concentrated using 10 kDa molecular weight cut-off Amicon Ultra centrifugal filter units (Merck, Burlington, MA, USA). Protein concentrations of both substrates were determined using the Qubit Protein Assay Kit prior to deglycosylation experiments.

### 4.3. Gene Cloning, Expression, and Purification

The coding sequence of EndoBI-1 (BLON_2468; EC 3.2.1.96) was amplified from *B. infantis* ATCC 15697 genomic DNA using gene-specific primers designed according to the Expresso Rhamnose Cloning and Expression System guidelines (Lucigen Corporation, Middleton, WI, USA) [41]. The forward primer (5′-CATCATCACCACCATCACGACGCCGTTTCTCCGACA-3′) incorporated the vector-homologous 6xHis tag sequence at the 5′ end, while the reverse primer (5′-GTGGCGGCCGCTCTATTAGGTCGCACTCAGTTGCTTCGG-3′) was designed for directional cloning into the pRham N-His Kan Vector. The amplified PCR product was cloned into the pre-linearized pRham N-His Kan Vector via Expressioneering Technology, an *in vivo* recombinational cloning strategy that does not require enzymatic treatment or ligation [33]. The construct was designed to include an N-terminal 6xHis affinity tag, while transmembrane regions and signal peptide sequences were omitted to facilitate soluble recombinant expression [21]. The integrity of the cloned insert was verified by colony PCR using the pRham Forward (5′-GCTTTTTAGACTGGTCGTAGGGAG-3′) and pETite Reverse (5′-CTCAAGACCCGTTTAGAGGC-3′) primers provided with the kit, followed by Sanger sequencing.

Recombinant EndoBI-1 was expressed in *E. cloni* 10G cells that were grown in LB medium with the addition of 30 µg/mL kanamycin. Protein expression was triggered by adding L-rhamnose to a final concentration of 0.1% (*w*/*v*) when the OD600 was between 0.2 and 0.8 and then incubating at 24 °C for 18 to 24 h. After expression, cells were collected by centrifugation and lysed through sonication in lysis buffer (50 mM NaH_2_PO_4_, 300 mM NaCl, pH 8.0). The His-tagged recombinant enzyme was purified from the cleared lysate using nickel-affinity chromatography and was eluted with a solution of 250 mM imidazole. Protein purity was evaluated through SDS-PAGE analysis on 4-12% gradient gels, and the enzyme concentration was measured using the Qubit Protein Assay Kit. The purified EndoBI-1 enzyme was stored at −80 °C until further use.

#### Verification of EndoBI-1 Activity Using Model Glycoprotein RNase B

RNase B, which contains only high-mannose-type *N*-glycans, was used as a model glycoprotein to confirm the enzymatic activity of recombinant EndoBI-1. 5 µg of RNase B was denatured by incubation at 95 °C for 5 min in Laemmli sample buffer. The denatured RNase B samples were then incubated for 1.5 h at 37 °C in a shaking incubator (200 rpm) with EndoBI-1 at a final enzyme concentration of 0.025 mg/mL in 0.02 M Na_2_HPO_4_ buffer (pH 5). Following incubation, all samples were run on 4-12% SDS-PAGE gels and stained with Coomassie Brilliant Blue for 30 min [41].

### 4.4. Deglycosylation of Glycoproteins

To characterize the *N*-glycans released from bLF and soy protein, glycoprotein samples (5 mg/mL) were independently deglycosylated using EndoBI-1 (0.025 mg/mL). The enzyme-to-substrate ratio was selected based on previously optimized conditions reported for EndoBI-1 activity on milk-derived glycoproteins [19]. Enzymatic reactions were carried out in 0.02 M Na_2_HPO_4_ buffer at pH 5, corresponding to the optimum pH for EndoBI-1 activity, at 37 °C for 3 h without prior denaturation of the protein substrates. Deglycosylation reactions for each substrate were performed in three independent replicates. The three replicate reactions were subsequently combined into a single pooled sample per substrate prior to procainamide labeling and HILIC-FLD-QTOF-MS/MS analysis. Following enzymatic incubation, the released *N*-glycans were recovered by protein precipitation using cold ethanol (4:1, *v*/*v*). Protein concentrations were determined using the Qubit Protein Assay Kit [19].

### 4.5. N-Glycans Profiling Using Hydrophilic Interaction Liquid Chromatography-Fluorescence Detection-Quadrupole Time-of-Flight Tandem Mass Spectrometry (HILIC-FLD-QTOF-MS/MS)

#### 4.5.1. Procainamide Labeling

Procainamide labeling of released *N*-glycans was performed with slight modifications to previously reported protocols [32]. Briefly, 25 μL of each glycan sample was mixed with 12.5 μL of procainamide hydrochloride solution (38.3 mg/mL in DMSO/acetic acid, 7:3, *v*/*v*) and 12.5 μL of picoline borane solution (44.8 mg/mL in DMSO/acetic acid, 7:3, *v*/*v*). The resulting reaction mixtures were incubated at 65 °C for 2 h to allow reductive amination to proceed.

#### 4.5.2. Purification of Procainamide-Labeled *N*-Glycans

For glycan clean-up, a small amount of cotton wool was inserted into a 10-100 μL pipette tip. The cotton-packed tips were conditioned by rinsing five times with 100% water, followed by washing with 85% ACN solution. Subsequently, 30 μL of procainamide-labeled *N*-glycan sample was mixed with 170 μL ACN to prepare the loading solution. The mixture was applied to the cotton-packed tip by repeated aspiration and dispensing (20-25 cycles). After sample loading, the tips were washed five times with 100 μL of 85/14/1 (*v*/*v*/*v*) ACN/water/trifluoroacetic acid (TFA), followed by five washes with 85/15 (*v*/*v*) ACN/water. The retained *N*-glycans were then recovered by elution through repeated pipetting (20-25 cycles) with 25 μL of 100% water. The resulting purified glycan samples were collected and prepared for subsequent analysis. After *N*-glycan release, the labeled glycans were purified then combined together before the profiling of the glycans was performed using HILIC-FLD-QTOF-MS/MS, providing a qualitative glycan profile.

#### 4.5.3. HILIC-FLD-QTOF-MS/MS Analysis of *N*-Glycans

Procainamide-labeled *N*-glycans were analyzed by hydrophilic interaction liquid chromatography (HILIC) using an Agilent 1200 Series HPLC system equipped with a fluorescence detector and coupled to a quadrupole time-of-flight mass spectrometer (timsTOF, Bruker Daltonics, GmbH, Bremen, Germany). Chromatographic separation was achieved on a Waters Glycan BEH Amide column (1.7 µm, 2.1 mm × 150 mm). The mobile phase system consisted of 100% ACN (solvent A) and 50 mM ammonium formate buffer at pH 4.4 (solvent B). A linear gradient was applied in which solvent A was decreased from 75% to 25% over 65 min at a constant flow rate of 0.25 mL/min. For each run, 40 μL of sample was injected. Fluorescence detection was performed with excitation and emission wavelengths set to 310 nm and 370 nm, respectively. Instrument control and data acquisition were carried out using HyStar 4.1 software (Bruker Daltonics, GmbH). Mass spectrometric analysis was performed on the timsTOF instrument operated strictly in QTOF mode, with the ion mobility (TIMS) function disabled. Ion source parameters were set as follows: capillary voltage 4.5 kV, source temperature 250 °C, nebulizer pressure 1.8 bar, and drying gas flow 7 L/min. MS data were acquired across an m/z range of 50–2800 at a scan rate of 0.5 Hz, ensuring sufficient spectral resolution for glycan compositional analysis. For MS/MS experiments, precursor ions were selected within a preferred *m*/*z* range of 500–2500. Singly charged ions and ions with undefined charge states were excluded, while ions carrying charge states from +2 to +5 were preferentially targeted. A group length of five spectra was applied for precursor selection, and active exclusion was enabled to minimize repeated fragmentation events and enhance overall glycan coverage.

The raw LC-QTOF-MS/MS data were first processed using the GlycoQuest algorithm in the ProteinScape software V4 (Bruker Daltonics). A search for glycan was carried out against the CarbBank glycan database with procainamide as the reducing end modification (reducing end mass: 219.173548 Da). Protonated ions (+H) of charge states +1 to +4 were considered for the searches. The precursor mass tolerance of 20 ppm was used while the MS/MS fragment ion tolerance was 0.05 Da. Fragment ions (b, c, y, z, cross-ring or 3CL) were used to search CID spectra. Only glycans with a GlycoQuest score of at least 10, a fragment coverage of at least 10% and an intensity coverage of at least 10% were automatically assigned. All glycan assignments generated by the computer were then checked manually by comparing the theoretical and experimental precursor masses, isotopic distribution, diagnostic fragment ions, chromatographic retention time, and glycan compositions through GlycoWorkBench. Consistent precursor mass accuracy and characteristic MS/MS fragmentation patterns were required for the assignment of any given mass. Glycans were characterized by their glycan composition using the Symbol Nomenclature for Glycans (SNFG). Only compositional isomers were obtained, as no structural or linkage isomers were performed. Identified *N*-glycans were annotated based on their mass spectral features using GlycoWorkBench software. Structural assignments were performed with reference to a glycan library containing the common *N*-glycan monosaccharides, including mannose, galactose, *N*-acetylglucosamine, fucose, *N*-acetylneuraminic acid, and *N*-glycolylneuraminic acid [32]. Every reported *N*-glycan composition was confirmed by MS/MS fragmentation data; accurate mass alone was not considered sufficient for structural assignment.

## 5. Conclusions

In the current research, recombinant EndoBI-1 was successfully produced in *E. coli* and purified to homogeneity as confirmed by SDS-PAGE analysis. The enzyme demonstrated *N*-glycan release activity on two structurally distinct glycoprotein substrates, bLF as a representative of animal-derived glycoproteins and soy protein as a representative of plant-derived glycoproteins under native, non-denaturing reaction conditions. HILIC-FLD-QTOF-MS/MS profiling of *N*-glycans showed differences in glycan composition depending on the substrate used: bLF yielded a structurally diverse array of *N*-glycan compositions encompassing high-mannose, complex-type, and sialylated structures, consistent with the established glycosylation profile of bLF. In contrast, soy protein produced high-mannose-type *N*-glycans, which corresponds to the typical glycosylation pattern of plant-derived storage proteins.

To our knowledge, this is the first report of EndoBI-1 activity on a plant-derived glycoprotein substrate characterized by HILIC-FLD-QTOF-MS/MS. These findings demonstrate the capacity of EndoBI-1 to release structurally intact *N*-glycans from the two glycoprotein substrates tested, one animal-derived and one plant-derived, under native, non-denaturing conditions, highlighting its potential as a biocatalytic tool for the preparative-scale production of bioactive *N*-glycans. The analytical technique adopted here, combining procainamide labeling with HILIC-FLD-QTOF-MS/MS, allows high-resolution structural characterization of released glycans and provides a robust platform for comparative glycomic studies across protein sources. Future work should address the functional characterization of the released *N*-glycans, a systematic comparison of EndoBI-1 with commercial deglycosylation enzymes under both native and denaturing conditions, extension of the substrate scope to additional plant and animal glycoproteins, and the potential of EndoBI-1 for preparative-scale glycan production.

## Figures and Tables

**Table 1:** Identification and validation of *N*-glycan compositions released from bLF and soy protein by recombinant EndoBI-1. Glycan compositions were initially assigned using the ProteinScape GlycoQuest algorithm and subsequently verified by manual inspection of precursor mass accuracy, chromatographic retention behavior, and diagnostic MS/MS fragment ions.

Bovine Lactoferrin							
Glycan Composition	Theoretical Mass (*m*/*z*)	Observed *m*/*z*	Mass Error	Mass Error (ppm)	z	Retention Time (min)	Identification Level
H3N3-proc	667.2976	667.2975	−0.0001	−0.15	2	21.5	MS1
H4N2-proc	646.784	646.7838	−0.0002	−0.31	2	23.5	MS1
H5N1-proc	626.2711	626.2707	−0.0004	−0.64	2	25.6	MS/MS
H4N3-proc	748.324	748.3236	−0.0004	−0.53	2	26.3	MS/MS
H3N3S1-proc	812.8453	812.8437	−0.0016	−1.97	2	27.8	MS/MS
H4N4-proc	849.8637	849.8622	−0.0015	−1.76	2	29.2	MS1
H6N1-proc	707.2975	707.2959	−0.0016	−2.26	2	30.0	MS/MS
H5N3-proc	829.3504	829.3499	−0.0005	−0.60	2	31.0	MS1
H4N3S1-proc	893.8717	893.8703	−0.0014	−1.62	2	31.3	MS/MS
H3N5S1-proc	1015.9247	1015.9222	−0.0025	−2.46	2	33.1	MS/MS
H5N2S1-proc	873.3584	873.3563	−0.0021	−2.40	2	33.5	MS/MS
H7N1-proc	788.3207	788.3219	0.0012	1.52	2	34.3	MS/MS
H5N3S1-proc	974.8982	974.8958	−0.0024	−2.46	2	36.1	MS/MS
H6N2S1-proc	954.3849	954.3812	−0.0037	−3.88	2	37.8	MS1
H8N1-proc	869.3503	869.3483	−0.002	−2.30	2	38.3	MS/MS
H5N3S2-proc	1120.4458	1120.4433	−0.0025	−2.23	2	40.6	MS1
H9N1-proc	950.3767	950.3759	−0.0008	−0.84	2	42.0	MS/MS
Soy protein							
H5N1-proc	626.2711	626.2688	−0.0023	−3.67	2	25.7	MS/MS
H6N1-proc	707.2975	707.2944	−0.0031	−4.38	2	30.5	MS/MS
H7N1-proc	788.3207	788.3194	−0.0013	−1.65	2	34.9	MS/MS
H8N1-proc	869.3503	869.3455	−0.0048	−5.52	2	39.0	MS/MS

MS1: Structure was proposed based on the precursor ion mass confirmed in MS1 mode.

**Table 2:** Comparison of *N*-glycan compositions released by EndoBI-1, from bLF and soy protein. The table represents a qualitative compositional inventory of the pooled *N*-glycan sample from each substrate. Each reported composition is supported by MS/MS fragmentation data. Deglycosylation reactions for each substrate were performed in three independent replicates; replicate reactions were pooled prior to MS analysis.

	EndoBI-1	
	bLF	Soy
**Number of *N*-glycans compositions**	17	4
**High mannose**	5	4
**Complex type**	5	0
**Antennary Fucosylation**	0	0
**Mono**	0	0
**Di**	0	0
**Sialylation** **(NeuAc)**	7	0
**Mono**	6	0
**Di**	1	0
**NeuGc content**	0	0

## Data Availability

The data presented in this study are available on request from the corresponding author.
